# Decreased Methylenetetrahydrofolate Reductase Activity Leads to Increased Sensitivity to *para*-Aminosalicylic Acid in Mycobacterium tuberculosis

**DOI:** 10.1128/AAC.01465-21

**Published:** 2022-01-18

**Authors:** Ji-fang Yu, Jin-tian Xu, Shan-shan Yang, Mei-na Gao, Hao-rui Si, Dong-yan Xiong, Jing Gu, Zhi-long Wu, Jie Zhou, Jiao-yu Deng

**Affiliations:** a Key Laboratory of Special Pathogens and Biosafety, Wuhan Institute of Virology, Center for Biosafety Mega-Science, Chinese Academy of Sciences, Wuhan, People’s Republic of China; b University of Chinese Academy of Sciences, Beijing, People’s Republic of China; c School of Chinese Materia Medica, Nanjing University of Chinese Medicine, Nanjing, People’s Republic of China; d Foshan Fourth People's Hospital, Foshan, People’s Republic of China; e Guangdong Province Key Laboratory of TB Systems Biology and Translational Medicine, Foshan Institude of Industrial Technology, Chinese Academic of Sciences, Foshan, People’s Republic of China

**Keywords:** *Mycobacterium tuberculosis*, methylenetetrahydrofolate reductase, Rv2172c, *para*-aminosalicylic acid, methionine

## Abstract

Tuberculosis (TB), caused by Mycobacterium tuberculosis, is one of the most fatal diseases in the world. Methylenetetrahydrofolate reductase (MTHFR) catalyzes the production of 5-methyltetrahydrofolate (5-CH_3_-THF), which is required for the *de novo* biosynthesis of methionine in bacteria. Here, we identified Rv2172c as an MTHFR in M. tuberculosis through *in vitro* and *in vivo* analyses and determined that the protein is essential for the *in vitro* growth of the bacterium. Subsequently, we constructed *rv2172c* R159N and L214A mutants in M. tuberculosis and found that these mutants were more sensitive to the antifolates *para*-aminosalicylic acid (PAS) and sulfamethoxazole (SMX). Combining biochemical and genetic methods, we found that *rv2172c* R159N or L214A mutation impaired methionine production, leading to increased susceptibility of M. tuberculosis to PAS, which was largely restored by adding exogenous methionine. Moreover, overexpression of *rv2172c* in M. tuberculosis could increase methionine production and lead to PAS resistance. This research is the first to identify an MTHFR in M. tuberculosis and reveals that the activity of this enzyme is associated with susceptibility to antifolates. These findings have particular value for antitubercular drug design for the treatment of drug-resistant TB.

## INTRODUCTION

Tuberculosis (TB), caused by Mycobacterium tuberculosis, is still one of the most lethal human diseases. M. tuberculosis is responsible for 10.0 million new cases of active TB and 1.5 million deaths annually (1). Since rifampicin was first used in a clinic in the 1968 (2), no new first-line antitubercular drugs have been identified over the past 50 years. By the 1990s, the emergence and spread of multidrug-resistant tuberculosis (MDR-TB) and extensively drug resistant tuberculosis (XDR-TB) has made the prevention and treatment of TB more challenging (3, 4). In order to fundamentally solve the problem of TB drug resistance, identifying new targets for drug design is essential. Additionally, it is equally important to further study the basic biology of M. tuberculosis, including the molecular mechanisms of M. tuberculosis sensitivity, resistance, and tolerance to existing antitubercular drugs.

*Para*-aminosalicylic acid (PAS) was the second antitubercular drug identified for clinical use, after streptomycin (5–7). PAS acts as a prodrug that activated by dihydropteroate synthase (DHPS) and dihydrofolate synthase (DHFS) before finally targeting dihydrofolate synthase (DHFR) in the folate biosynthesis pathway (6, 7). Sulfonamides were also the earliest identified antibacterial drugs (8). These compounds target the DHPS, which catalyzes the addition of dihydropterin diphosphate to *p*-aminobenzoic acid (*p*ABA) (8–10). As structural analogs of *p*ABA, sulfonamides compete with *p*ABA, leading to the waste of dihydropterin diphosphate and the blockage of dihydropteroate biosynthesis (8–10). Sulfamethoxazole (SMX; a kind of sulfonamide) and PAS were both important components in early TB chemotherapies, especially PAS (5). In later years, the introduction of more potent and less toxic antitubercular drugs regimens diminished their usage. With the emergence of MDR-TB and XDR-TB, PAS has returned to clinical application (11, 12). Moreover, SMX has been receiving increasing attention as a treatment for drug-resistant TB (13–15). PAS and SMX are both antifolates that inhibit *de novo* folate biosynthesis in bacteria (5, 8). Folate provides an important one-carbon for bacteria and is crucial for the *de novo* synthesis of methionine, purines, and deoxythymidine monophosphate (dTMP) (16). In addition, methionine is a potent antagonist of PAS (17, 18).

As the lynchpin of folate downstream metabolism, methylenetetrahydrofolate reductase (MTHFR) catalyzes the NAD(P)H-dependent reduction of 5,10-methylenetetrahydrofolate (5,10-CH_2_-THF) to 5-methyltetrahydrofolate (5-CH_3_-THF) (19). This reaction is the sole source of bacterial methyltetrahydrofolate (20), which provides one-carbon units for the synthesis of methionine and maintains the recycling and homeostasis of tetrahydrofolate. MTHFR is encoded by the *metF* gene in Escherichia coli. Previous research has shown that E. coli MTHFR has NADH-dependent reductase activity and uses either flavin adenine dinucleotide (FAD) or flavin mononucleotide (FMN) as a source of reducing equivalents for the reduction of 5,10-CH_2_-THF to 5-CH_3_-THF (19, 21, 22). MTHFR’s catalytic domain contains an α_8_β_8_ barrel (23). Mutation of Phe223 decreases both substrate and NADH binding (23, 24), while Asp120 mutation reduces MTHFR’s affinity for 5-CH_3_-THF (25), and Glu28 (proton donor or acceptor) mutation abolishes the enzymatic activity of MTHFR (25, 26). A nucleotide phosphate-binding region (NPBR), located from amino acids 165 to 172, was also shown to be important for enzyme activity due to FAD binding (23). Although the properties and crystal structure of E. coli MTHFR have been extensively explored (23–27), MTHFR in M. tuberculosis has not been uniquely identified or annotated. A previous review article revealed the important clue that Rv2172c might be an atypical MetF homologue, because its predicted structure significantly matched the crystal structure of MetF from Thermus thermophilus (28). Unfortunately, there were no further experimental data following this observation. Here, using E. coli W3110 and M. tuberculosis H37Ra as bacterial models, we carried out a series of experiments *in vitro* and *in vivo* to characterize whether Rv2172c was the MTHFR in M. tuberculosis. Additionally, we aimed to reveal the relationship between the activity of Rv2172c and the sensitivity of M. tuberculosis to antifolate PAS.

## RESULTS

### The predicted three-dimensional structure of Rv2172c has relatively high similarity to the structure of E. coli MTHFR.

**FIG 1:**
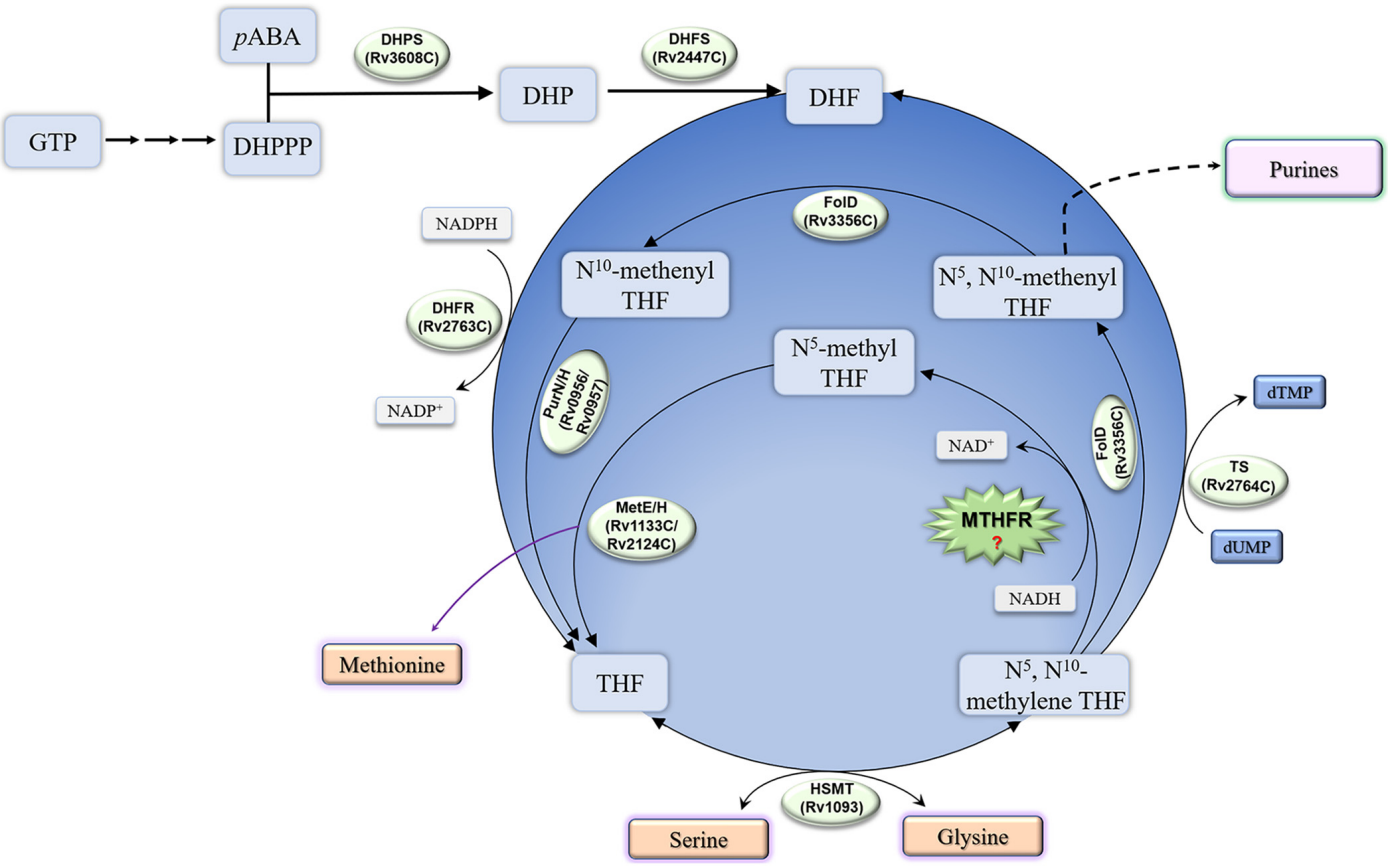
MTHFR remains unknown in M. tuberculosis. MTHFR, 5,10-methylenetetrahydrofolate reductase; DHPS, dihydropteroate synthase; DHFS, dihydrofolate synthase; DHFR, dihydrofolate reductase; TS, thymidylate synthase; MetE, cobalamin-independent homocysteine transmethylase; MetH, cobalamin-dependent methionine synthase; FolD, bifunctional 5,10-methylenetetrahydrofolate dehydrogenase/cyclohydrolase; PurH, bifunctional AICAR formyltransferase/IMP cyclohydrolase; PurN, phosphoribosylglycinamide formyltransferase; HSMT, serine hydroxymethyltransferase.

**FIG 2:**
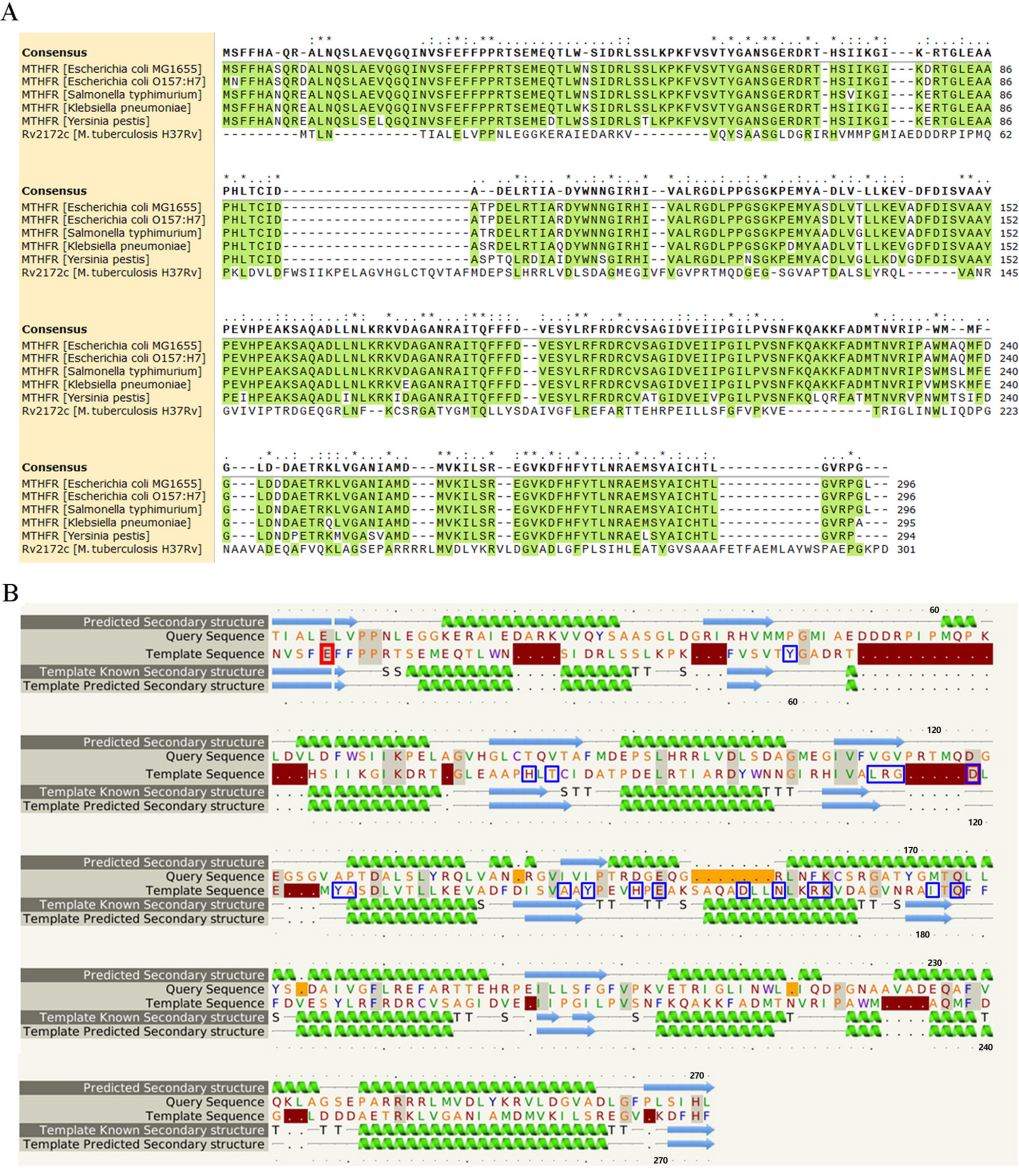
The homology of Rv2172c from M. tuberculosis with MTHFR in E. coli. (A) Rv2172c and several Enterobacter MTHFR primary sequence alignment. (B) Prediction of Rv2172c protein secondary structure using Phyre 2. Each row from the top to the bottom is Rv2172c predicted secondary structure, Rv2172c primary sequence, template E. coli MTHFR primary sequence, template E. coli MTHFR known secondary structure, and template E. coli MTHFR predicted secondary structure (as the control to test the confidence of prediction). Identical residues in the alignment are highlighted with a gray background. Residues marked by blue boxes are the residues of E. coli MTHFR interacting with FAD, as claimed in previous studies (23, 24). Residues marked by red boxes are catalytic residues from the Catalytic Site Atlas (CSA).

**FIG 3:**
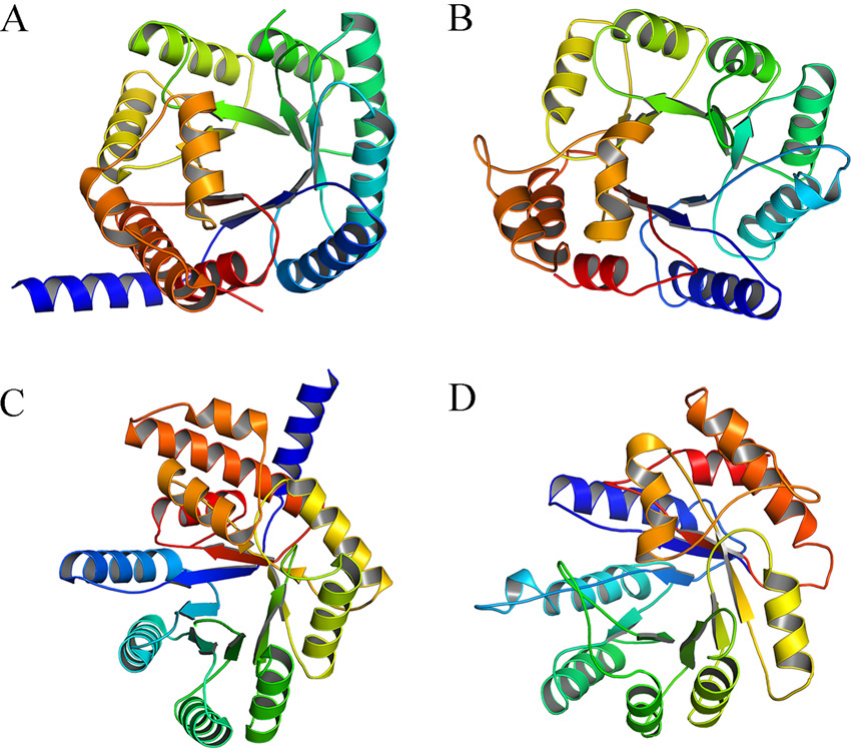
The predicted three-dimensional (3D) structure of Rv2172c compared with the known structure of E. coli MetF. Rv2172c was modeled based on the best-matched template from Thermus thermophilus MTHFR (PDB entry 3APT). (A) Front view of MetF, (B) front view of Rv2172c, (C) side view of MetF, and (D) side view of Rv2172c.

MTHFR is a crucial enzyme in folate downstream metabolism, but it remains unidentified in M. tuberculosis (Fig. 1). We aligned the primary sequence of Rv2172c with MTHFR homologues from several Enterobacter species. The results showed that the enzymes from Enterobacter had significant primary sequence homology, while Rv2172c showed very low similarity to these (Fig. 2A). Considering the differences between these two genera in terms of GC content, protein translation, and direction of bacteria evolution, we predicted the secondary structure of Rv2172c using Phyre 2 as previously reported (29). We found that the high-confidence template was a mutated E. coli MTHFR Ala177Val (Fig. 2B; see also Fig. S1A in the supplemental material). In addition, most matched templates all had methylenetetrahydrofolate reductase activity (Fig. S1B). Next, structural modeling of Rv2172c revealed a higher similarity with MTHFR from E. coli (PDB entry 1ZP3) than with primary sequence homology (Fig. 3).

### Rv2172c complemented the growth defect of the E. coli Δ*metF* strain.

**FIG 4:**
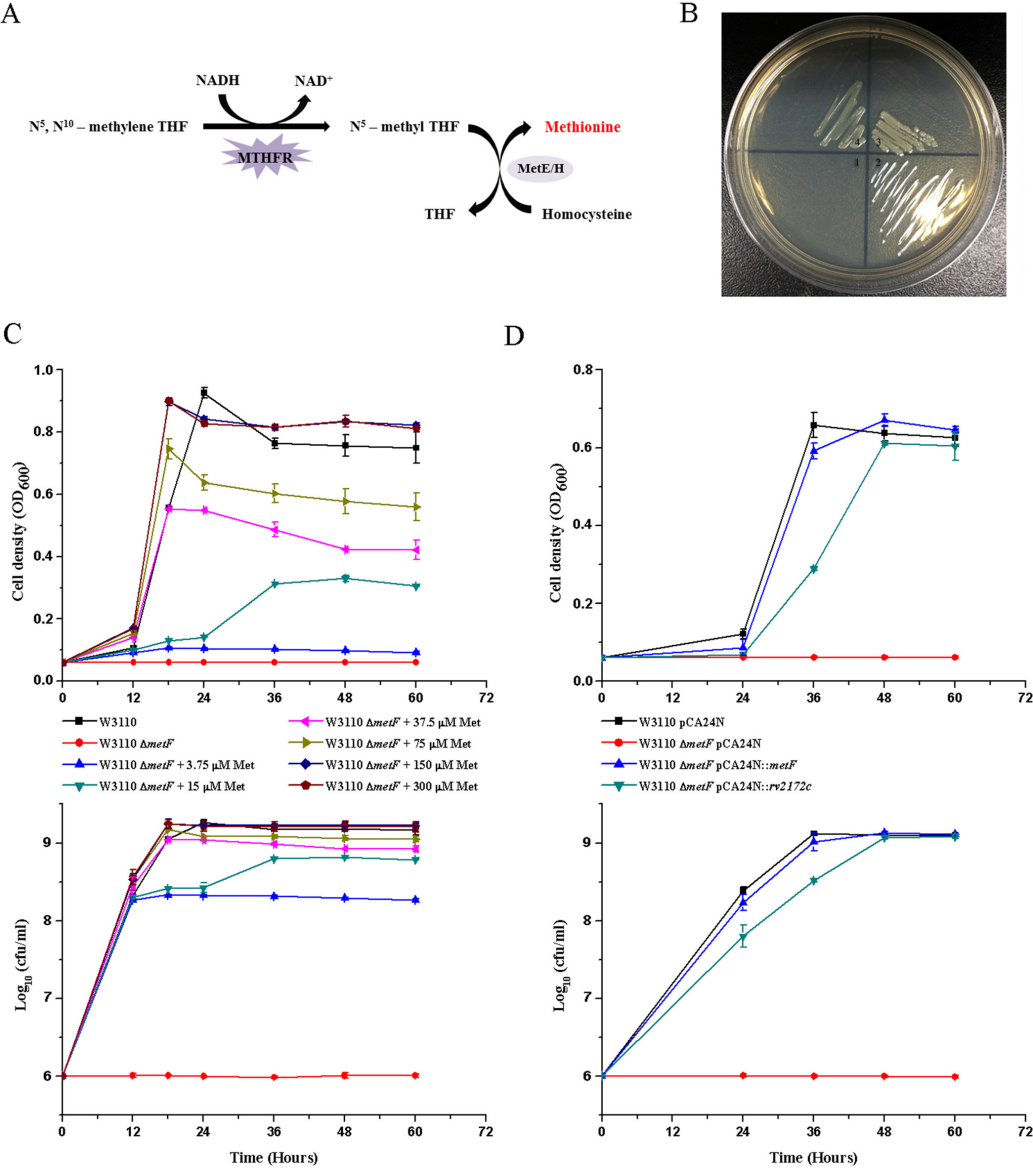
The MTHFR activity of Rv2172c *in vivo*. (A) MTHFR reaction downstream of the methionine synthesis pathway. (B) E. coli W3110 Δ*metF* strains harboring (1) no vector, (2) empty pCA24N vector, (3) pCA24N::*metF*, and (4) pCA24N::*rv2172c* were inoculated on solid minimal medium. (C) E. coli W3110 Δ*metF* was cultured on minimal medium containing different concentrations of methionine (Met). E. coli W3110 was used as a control. Optical density at 600 nm (OD_600_) (upper) and log_10_ CFU (lower) were measured at appropriate times. (D) E. coli W3110 Δ*metF* strains harboring empty pCA24N vector, pCA24N::*metF*, or pCA24N::*rv2172c* were cultured on minimal medium. E. coli W3110 pCA24N was the control background. OD_600_ (up) and log_10_ CFU (down) were measured at appropriate times. The experiments were performed using three biological replicates. The standard deviations (SDs) are indicated by the error bars, and the data are reported as mean ± SD.

As shown in Fig. 4A, MTHFR converts 5,10-CH_2_-THF to 5-CH_3_-THF, and then methionine synthase (MetH/E) catalyzes the transfer of a methyl group from 5-CH_3_-THF to homocysteine to form methionine (19, 30). This MTHFR activity is thus crucial for methionine biosynthesis in bacteria, and deleting MTHFR-encoding genes leads to a methionine auxotroph phenotype. To test our putative M. tuberculosis MTHFR, we knocked out the MTHFR-coding gene *metF* in E. coli, which resulted a methionine auxotroph phenotype, as expected (Fig. 4B and C). The growth defect caused by *metF* deletion could be rescued by adding exogenous methionine *in vitro*, and the bacterial growth was methionine dose dependent (Fig. 4C). To explore the physiological role of *rv2172c* in M. tuberculosis and verify whether Rv2172c has MTHFR activity *in vivo*, we tested the ability of Rv2172c to rescue the methionine auxotroph phenotype of the E. coli W3110 Δ*metF* strain. As shown in Fig. 4B and D, transformation with pCA24N::*rv2172c* or pCA24N::*metF* in the E. coli W3110 Δ*metF* strain both conferred growth in cultures on E medium without exogenous methionine addition.

### Rv2172c catalyzes 5,10-methylenetetrahydrofolate to produce 5-methyltetrahydrofolate in the presence of NADH.

**FIG 5:**
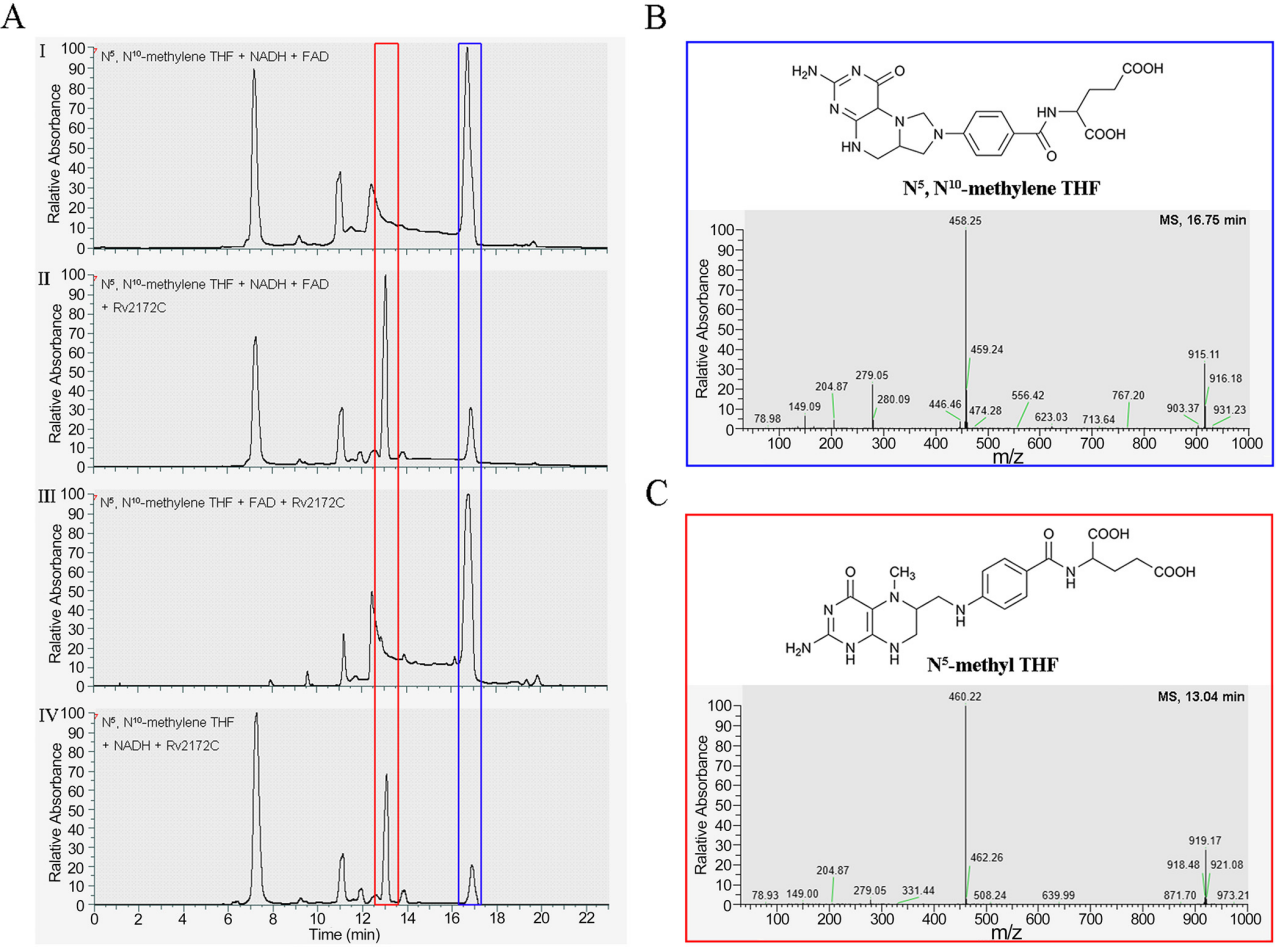
The reductase activity of Rv2172c by high-performance liquid chromatography–mass spectrometry (HPLC-MS). (A) HPLC analysis from different reaction systems containing (I) 5,10-CH_2_-THF, NADH, and FAD, (II) 5,10-CH_2_-THF, NADH, FAD, and Rv2172c; (III) 5,10-CH_2_-THF, FAD, and Rv2172c; and (IV) 5,10-CH_2_-THF, NADH, and Rv2172c. (B) The substrate in reaction system 2, 5,10-CH_2_-THF, was identified based on an MS peak at 458.25 *m/z*. (C) The product in reaction system 2, 5-CH_3_-THF, was identified based on an MS peak at 460.22 *m/z*.

Subsequently, we designed five reaction systems to assess the reductase activity of purified Rv2172c *in vitro* (see Fig. S2, inset table). MTHFR could utilize 5,10-CH_2_-THF (Fig. S2A) as a substrate to produce 5-CH_3_-THF (Fig. S2B). To identify the product formed in these reaction mixtures, high-performance liquid chromatography–mass spectrometry (HPLC-MS) analysis was performed. The base peak chromatogram from reaction 2 (Fig. 5A, II) showed the emergence of a new peak (at ∼13 min, red frame), which was identified as the product 5-CH_3_-THF based on the MS peak at 460.22 *m/z* (Fig. 5C). The substrate 5,10-CH_2_-THF (*m/z *= 458.27) in this reaction in the base peak chromatogram (at ∼16.8 min, blue frame) was also confirmed by MS analysis (Fig. 5B). When Rv2172c was absent, this reaction did not proceed (Fig. 5A, I). This protein had an NADH-dependent, but FAD-nondependent, reductase activity (Fig. 5A, III and IV). This was different from MetF of E. coli, but consistent with the MTHFR in Mycobacterium smegmatis (31). Additionally, a few FAD-interacting amino acids from MetF (23, 24) were found in similar locations in Rv2172c (Fig. 2B).

### The Rv2172c L214A and Rv2172c R159N mutants in M. tuberculosis showed reduced methionine production and growth defects.

We next tried to knock out *rv2172c* in M. tuberculosis by supplementing exogenous methionine into the growth medium but failed (data not shown). The original copy of the *rv2172c* gene on the M. tuberculosis chromosome could be deleted only in the presence of another copy of *rv2172c* introduced using a recombinant plasmid. Thus, we changed our tactic for constructing mutant strains that harbored point mutations in the *rv2172c* gene (Table S1). To achieve this, we first introduced the mutant genes into M. tuberculosis by using the recombinant plasmid pMV361 and then deleted the original copy of the *rv2172c* gene in the chromosome. The corresponding “Rv2172c (WT)” strain was also constructed through the same procedure; in this case, an intact *rv2172c* gene was introduced into the bacterium by using the same plasmid.

**FIG 6:**
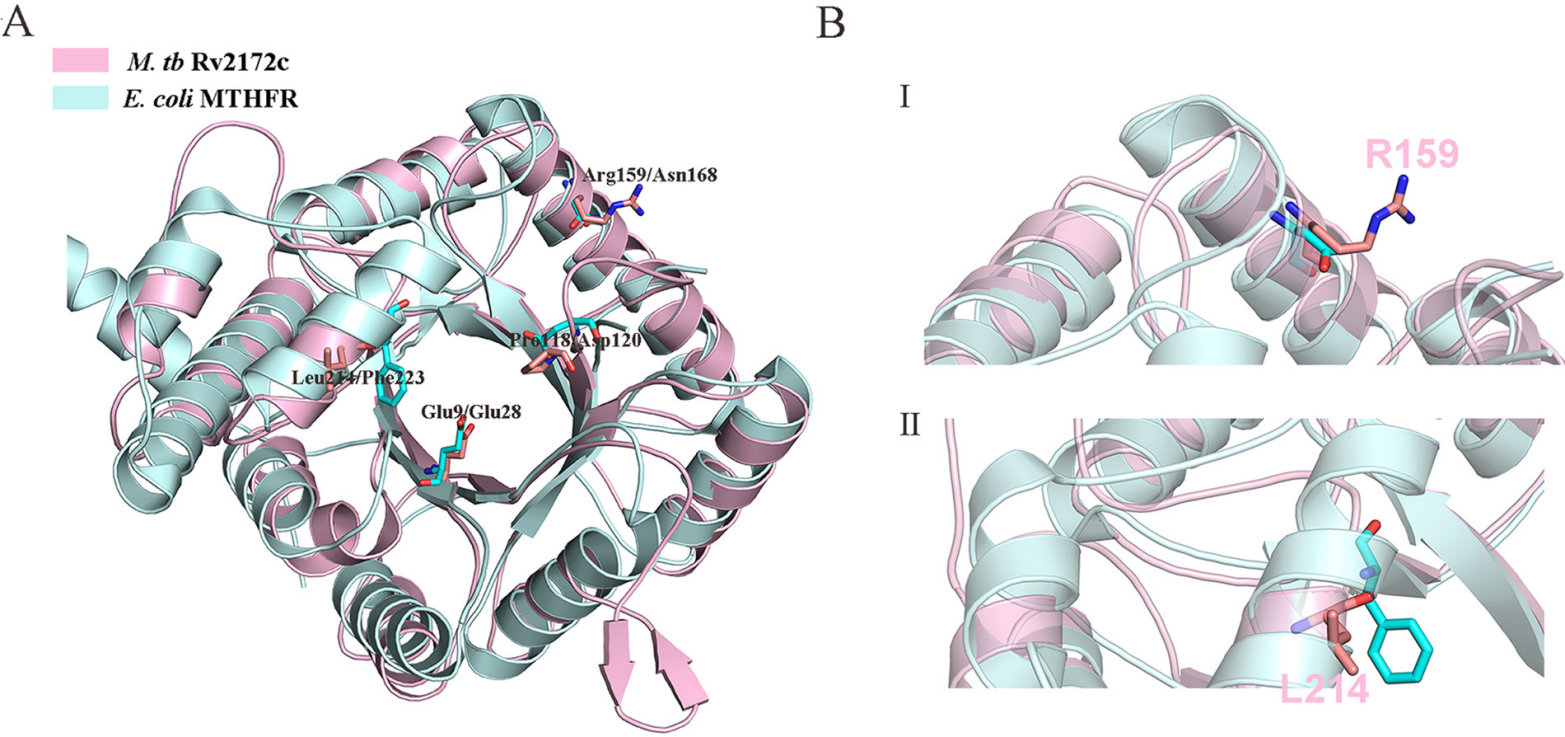
Comparison between a modeled structure of Rv2172c and the known structure of E. coli MTHFR. Rv2172c was modeled based on E. coli MetF (PDB entry 1ZP3). (A) Superimposition of these two structures highlights the high structural similarity at the level of the overall fold. Close-up view of the active site predicted from previous studies, including catalytic residues such as Glu9/Glu28, Pro118/Asp120, Arg159/Asn168, and Leu214/Phe223 (M. tuberculosis Rv2172c/E. coli MetF). (B) Representation of the catalytic residues (I) R159 and (II) L214.

To select suitable mutation sites, a modeled three-dimensional (3D) structure of Rv2172c (pink) was superimposed on the E. coli MTHFR structure (PDB entry 1ZP3) (blue). As shown in Fig. 6A, the known key catalytic residues (Glu28, Asp120, and Phe223) in E. coli MTHFR and their corresponding sites in Rv2172c were mapped. The center of the region from amino acids165 to 172 in E. coli MTHFR, Asn168 (Arg159 in Rv2712c), was also utilized. We tried to mutate these four amino acid residues in *rv2712c*, but successfully constructed only two mutants (*rv2172c* L214A and R159N) (Fig. 6B).

**FIG 7:**
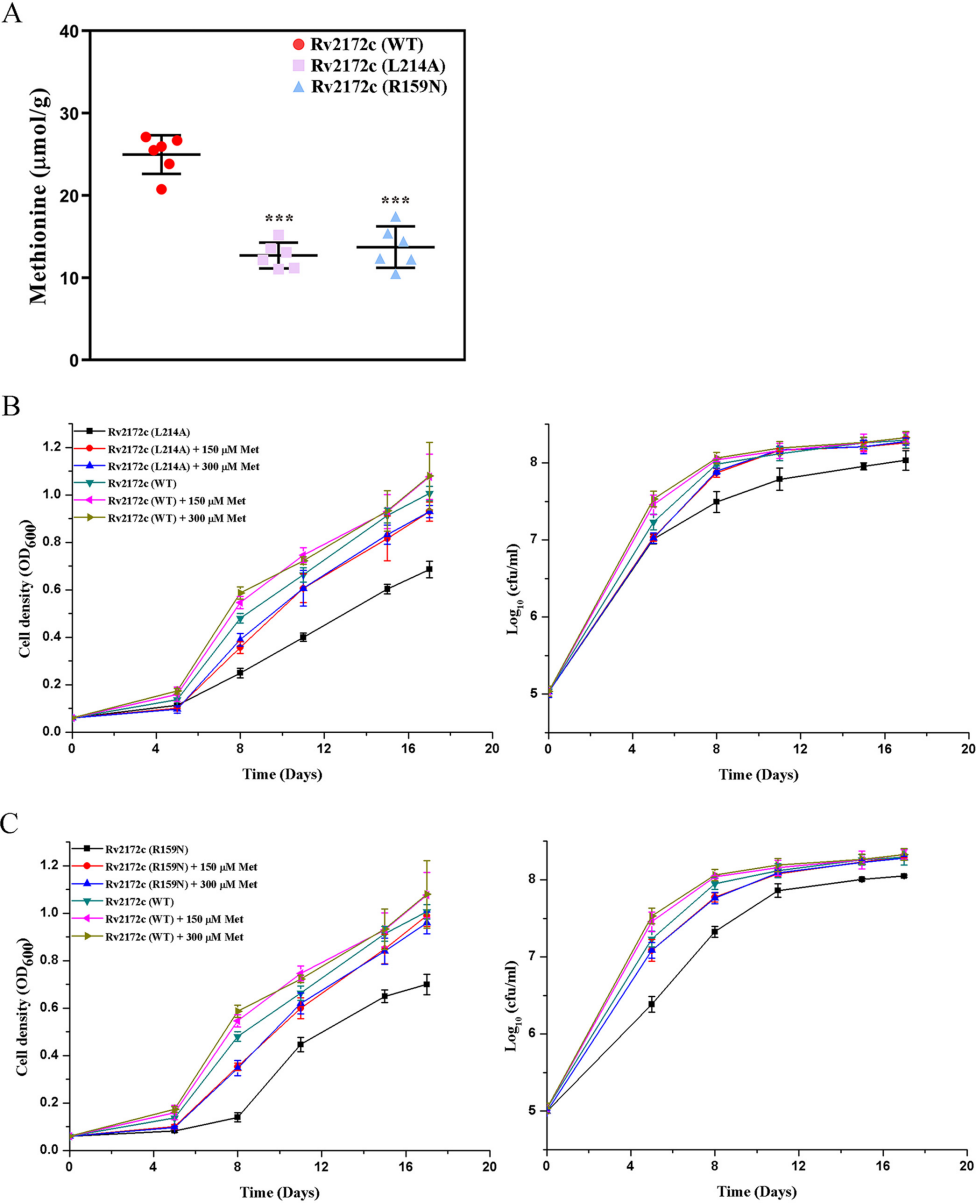
Assessment of the constitutive synthesis of methionine in *rv2172c* mutants. (A) The constitutive synthesis of methionine in *rv2172c* mutant cells was measured by ultraperformance liquid chromatography-tandem mass spectrometry (UPLC-MS/MS). Cell-associated methionine was extracted from Rv2172c (WT), Rv2172c (L214A), and Rv2172c (R159N) strains and quantified as described in Materials and Methods. The experiments were performed using six biological replicates. *P* values were calculated using *t* tests. ***, *P* < 0.001. (B) Growth curves for Rv2172c (WT) and Rv2172c (L214A) in 7H9 (with OADC), in the presence of 150 μM or 300 μM methionine (Met). (C) Growth curves of Rv2172c (WT) and Rv2172c (R159N) in 7H9 (with OADC) with 150 μM or 300 μM methionine (Met). Rv2172c (WT), H37Ra Δ*rv2172c* pMV361::*rv2172c*; Rv2172c (L214A), H37Ra Δ*rv2172c* pMV361::*rv2172c* (L214A); Rv2172c (R159N), and H37Ra Δ*rv2172c* pMV361::*rv2172c* (R159N). OD_600_ (left) and log_10_ CFU (right) were measured at appropriate times. No addition of methionine was used as a control condition. Data represent the mean ± SD.

We speculated that Arg159 or Leu214 were important for the MTHFR activity of Rv2172c and that R159N or L214A mutation might disturb methionine synthesis in M. tuberculosis. To detect the methionine content in these mutants, ultraperformance liquid chromatography-tandem mass spectrometry (UPLC-MS/MS) analysis was performed. As shown in Fig. 7A, the methionine content of Rv2172c R159N or L214A was significantly lower than that of the “Rv2172c (WT)” strain.

Without adding exogenous methionine, the E. coli *metF* knockout strain showed no growth on minimal medium. The Rv2172c R159N or L214A mutants also showed some degree of growth defect compared to the “Rv2172c (WT)” strain (Fig. 7B and C).

### Rv2172c L214A and Rv2172c R159N mutants showed increased susceptibility to PAS and SMX.

Previous studies have shown that methionine is a potent antagonist of PAS (17, 18). Since the mutations L214A and R159N led to decreased methionine production in M. tuberculosis, we speculated that these two mutations might affect susceptibility to PAS. SMX and PAS are both commonly used antifolates, and MTHFR is a crucial enzyme in folate metabolism. Therefore, the susceptibilities of these two mutants to PAS and SMX were determined using a MIC assay. As shown in Table 1, the MICs of PAS and SMX in the Rv2172c L214A mutant were 20-fold and 32-fold lower, respectively, than that of the “Rv2172c (WT)” strain. The MICs of PAS and SMX in the Rv2172c R159N mutant were 20-fold and 4-fold lower, respectively, than that of the “Rv2172c (WT)” strain. In the “Rv2172c (WT)” strain (H37Ra Δ*rv2172c* pMV361::*rv217c*), the chromosomal copy of *rv2172c* was replaced by an intact *rv2172c* under the control of the Hsp60 promoter, resulting in overexpression of the gene and decreased susceptibility to PAS compared to that of the H37Ra strain.

**TABLE 1:** Replacement of Rv2172c with Rv2172c L214A and R159N confers sensitivity to the antifolates PAS and SMX in M. tuberculosis H37Ra

Strain^*a*^	MIC (μg · mL^−1^) for:	
	PAS	SMX
Rv2172c (WT)	0.2	100
Rv2172c (L214A)	0.01	3.125
Rv2172c (R159N)	0.01	25

a Rv2172c (WT), H37Ra Δ*rv2172c* pMV361::*rv2172c*. Rv2172c (L214A), H37Ra Δ*rv2172c* pMV361::*rv2172c* (L214A). Rv2172c (R159N), H37Ra Δ*rv2172c* pMV361::*rv2172c* (R159N).

### Exogenous methionine rescues the growth defect of Rv2172c L214A and Rv2172c R159N, reversing their increased sensitivities to PAS.

**FIG 8:**
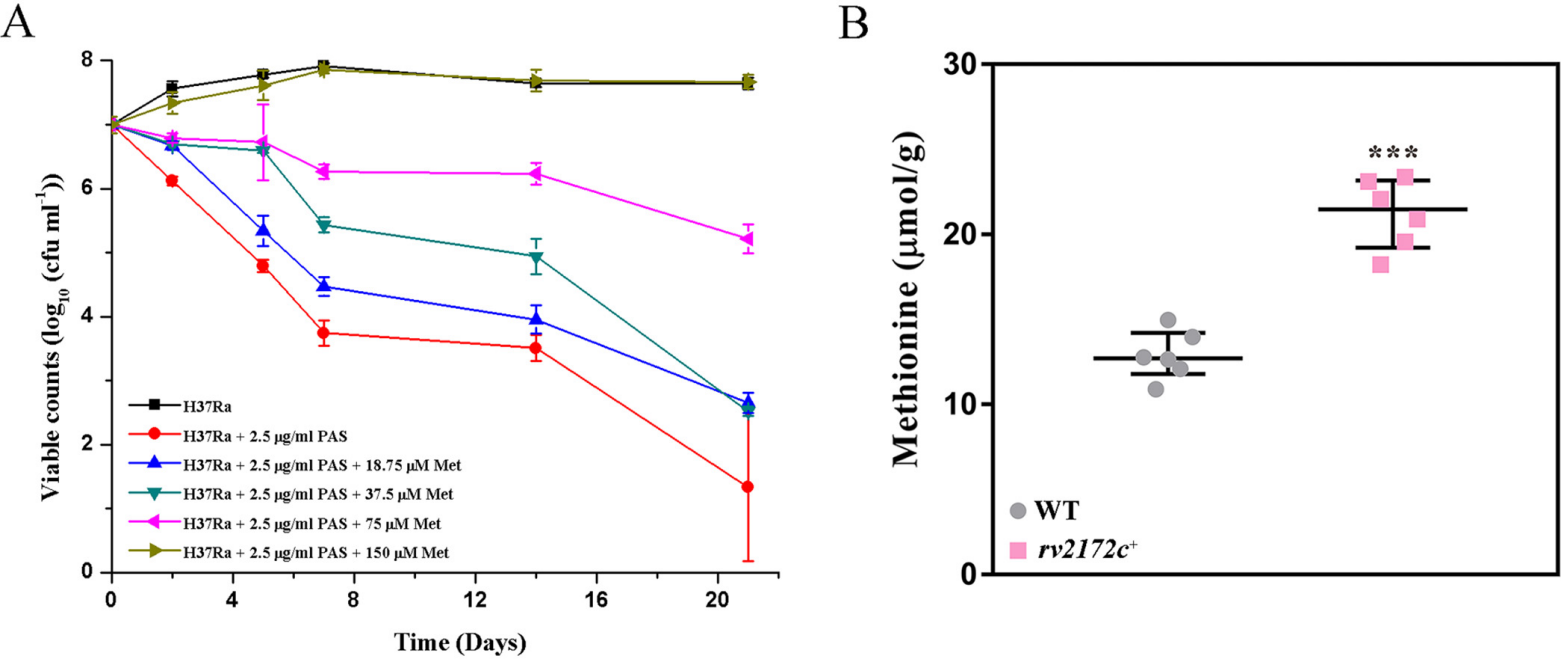
Methionine antagonize PAS and the quantitative detection of methionine by UPLC-MS/MS in *rv2172c* overexpression strains. (A) H37Ra was cultured in 7H9 broth (with OADC), containing 2.5 μg/mL PAS and different concentrations of methionine (Met). No PAS addition was used as a control. Experiments were performed using three biological replicates. Data represent the mean ± SD. (B) The synthesis of methionine in the *rv2172c* overexpression strains was detected via UPLC-MS/MS. Cell-associated methionine was extracted from WT (H37Ra pMV261) and *rv2172c*^+^ (H37Ra pMV261::*rv2172c*) strains and quantified as described in Materials and Methods. The experiments were performed using six biological replicates. *P* values were calculated using *t* tests. ***, *P* < 0.001.

In order to determine whether the growth defect of these two mutants was caused by reduced methionine production *in vivo*, we added two concentrations of methionine to our growth medium and compared the growth curves of Rv2172c R159N and Rv2172c L214A with that of the “Rv2172c (WT)” strain. As shown in Fig. 7B and C, the growth defect of these mutants could be largely restored by adding exogenous methionine. Moreover, 150 μM or 300 μM methionine worked similarly in this context. This indicated that 150 μM methionine might be sufficient to support the *in vitro* growth of these two mutants. In contrast, 150 μM methionine could completely reverse the bactericidal effect of PAS (Fig. 8A). Thus, the MICs of PAS in these two Rv2172c mutants and the “Rv2172c (WT)” strain in the presence of 150 μM methionine were tested. As shown in Table 2, in the presence of methionine, both of these two mutants became highly resistant to PAS.

**TABLE 2:** Methionine reverts the sensitivity of Rv2172c L214A and R159N to PAS in M. tuberculosis H37Ra

Strain^*a*^	MIC (μg · mL^−1^) for:	
	PAS	PAS added with 150 μM Met
Rv2172c (WT)	0.2	1.6
Rv2172c (L214A)	0.01	0.4
Rv2172c (R159N)	0.01	0.4

a Rv2172c (WT), H37Ra Δ*rv2172c* pMV361::*rv2172c*. Rv2172c (L214A), H37Ra Δ*rv2172c* pMV361::*rv2172c* (L214A). Rv2172c (R159N), H37Ra Δ*rv2172c* pMV361::*rv2172c* (R159N).

### Overexpressing *rv2172c* in M. tuberculosis increased the production of methionine but decreased M. tuberculosis sensitivity to PAS.

To further explore how mutations in *rv2172c* changed the sensitivity to PAS in M. tuberculosis, *rv2172c* and other methionine synthase coding genes (*metE* and *metH*) were overexpressed in M. tuberculosis. The results showed that overexpressing all of these genes led to PAS resistance in M. tuberculosis (Table 3). Correspondingly, we observed an obvious increase in methionine production in the *rv2172c* overexpression strain compared to the wild-type (WT) strain (Fig. 8B).

**TABLE 3:** Overexpression of primary genes that participate in methionine biosynthesis confers PAS resistance in M. tuberculosis H37Ra

Strain^*a*^	MIC (μg · mL^−1^) of PAS
WT	0.02
*MetE* ^+^	0.1
*MetH* ^+^	0.1
*rv2172c* ^+^	0.2

a WT, H37Ra pMV261. *MetE*^+^, H37Ra pMV261::*metE*. *MetH*^+^, H37Ra pMV261::*metH*. *rv2172c*^+^, H37Ra pMV261::*rv2172c*.

### Mutation analysis of *rv2172c* in M. tuberculosis.

We analyzed a library of 6,568 M. tuberculosis genomes from isolates classified as clinical (*n* = 6,310) and environmental or other (*n* = 258) from the National Center for Biotechnology Information (NCBI) database (Table S2). We found that the summed rate of nonsynonymous, indel, and nonsense single-nucleotide polymorphisms (SNPs) in *rv2172c* was very low (∼1%) in both the clinical and environmental isolates, and the major mutations identified in the environment isolates were synonymous mutations (Table 4). This indicated that the primary sequence of Rv2172c was relatively conserved.

**TABLE 4:** Mutation rates of *rv2172c* among 6,568 M. tuberculosis genomes

Mutation type	Rate (%) in:	
	Clinic	Environment
All types	1.49	8.14
Synonymous SNP	0.16	5.04
Nonsynonymous SNP	1.08	1.16
Indel or nonsense SNP	0.03	0.00

To further investigate the potential clinical relevance of *rv2172c* and PAS susceptibility, mutations of *rv2172c* and 3 other genes related to PAS resistance, *folC* (32, 33), *thyA* (32, 34, 35), and *ribD* (7, 32), were characterized by analyzing the whole-genome sequence of 52 PAS-resistant clinical isolates (Table S3). As shown in Table 5, the mutation rates of *folC* (40.38%), *thyA* (34.62%), and *ribD* (3.85%) were all within a normal fluctuation compared to the previous studies (32, 34). To our surprise, the mutation rate of *rv2172c* in the 52 PAS-resistant isolates was 9.62%, even higher than that of *ribD*. Two specific mutations were identified, T120P (5.77%) and M172V (3.85%). Thus, we further analyzed the mutation of *rv2172c* in 66 PAS-sensitive clinical isolates presented in previous work (Table S3). The mutation of *rv2172c* was found in one of the 66 PAS-sensitive isolates. The rate of this mutation, M172V (1.51%; 1/66), was lower in the PAS-sensitive isolates than that in the PAS-resistant isolates. In addition, the T120P mutation was not found in the 66 PAS-sensitive isolates.

**TABLE 5:** Gene mutation analysis in 52 PAS-resistant M. tuberculosis clinical isolates

Gene	Mutation type	Count	
		No.	%
*folC*		21	40.38
	E40G	9	17.31
	S150G	6	11.54
	I43T	2	3.85
	E153A	1	1.92
	E153G	1	1.92
	S98G	1	1.92
	A420V	1	1.92
*thyA*		18	34.62
	T202A	12	23.08
	S215F	1	1.92
	T26P	1	1.92
	H75N	1	1.92
	*thyA-dfrA* deletion	2	3.85
	*thyA* deletion	1	1.92
*rv2172c*		5	9.62
	T120P	3	5.77
	M172V	2	3.85
*ribD*		2	3.85
	^−11^G>A	2	3.85

## DISCUSSION

Although it is well known that 5-CH_3_-THF is an essential cofactor for bacterial methionine synthase, the enzyme catalyzing the production of 5-CH_3_-THF in M. tuberculosis has not yet been identified or characterized. Here, we show that *rv2172c* encodes an MTHFR in M. tuberculosis. Previously, Umesh et al. showed that there were two copies of MTHFR in M. smegmatis, MSMEG_6596 and MSMEG_6649 (31). Deletion of the major MTHFR encoding gene (*mseg_6596*) caused a partial methionine auxotroph in M. smegmatis, indicating they might be jointly essential for methionine biosynthesis. In M. tuberculosis, however, Rv2172c seems to be the only copy of an MTHFR, since *rv2172c* is essential for the *in vitro* growth of this bacterium. Although the MTHFR encoding gene *metF* was also shown to be essential for the *in vitro* growth of E. coli on minimum medium, an *metF* deletion mutant was successfully constructed by supplementing the growth medium with exogenous methionine. However, the original copy of *rv2172c* on the M. tuberculosis chromosome could be deleted only in the presence of another copy of *rv2172c*, in this case introduced by a recombinant plasmid. Otherwise, not a single transductant was obtained on 7H10 plates supplemented with 10% oleic acid-albumin-dextrose-catalase (OADC) and sufficient methionine. All of these data suggest that either Rv2172c has another yet unknown important function in addition to being an MTHFR, or that 5-CH_3_-THF participates in another essential physiological process in M. tuberculosis in addition to methionine biosynthesis, which merits further investigation.

PAS and SMX are two commonly used antifolates, and both are effective against M. tuberculosis (11–15). In the 1950s, it was found that methionine could antagonize the growth inhibition effect of PAS on M. tuberculosis (17, 18). Recently, Howe et al. showed that this antagonism could be abolished by disrupting *para*-aminobenzoic acid biosynthesis (36). Besides, the susceptibility to sulfonamides in M. tuberculosis was also shown to be associated with methionine, since deleting *metH* led to increased sensitivity to sulfonamides (37). These findings indicated that disturbing methionine synthesis might affect susceptibility to PAS and SMX in M. tuberculosis. As is well known, the pharmacodynamics of PAS and SMX are quite different. Here, we focused more on PAS, which is presently used as a second-line antituberculosis drug. Theoretically, there are multiple ways to disrupt methionine biosynthesis. Here, we show that decreasing methylenetetrahydrofolate reductase activity leads to decreased production of methionine in M. tuberculosis and hence makes this bacterium more sensitive to PAS. Methionine is an essential amino acid for humans, who have to ingest it from their diet. Bacteria, including M. tuberculosis, have a *de novo* synthesis pathway for methionine, and disrupting this usually causes a methionine auxotroph phenotype. Moreover, Berney et al. demonstrated that blocking methionine biosynthesis in M. tuberculosis rendered this pathogen exquisitely sensitive to death in mice (38). Thus, the methionine biosynthesis pathway seems to be an attractive target for designing new anti-TB drugs, especially given the global crisis of drug-resistant M. tuberculosis.

As a key enzyme responsible for producing a crucial cofactor required for methionine biosynthesis in M. tuberculosis, Rv2172c itself seems to be a good target for designing new anti-TB drugs. It is essential for the *in vitro* growth of M. tuberculosis and might also be important for the survival of this bacterium in a host, which merits further investigation. The fact that decreasing the activity of Rv2172c makes M. tuberculosis more sensitive to PAS and SMX implies that inhibitors of this protein can be used in combinations with other existing drugs. Although humans also have an MTHFR (39), considerable structural differences have been observed between it and Rv2172c (see Fig. S3 in the supplemental material).

Due to its essentiality for bacterial growth, the mutation rate of *rv2172c* is rather low (1.49%) in clinical isolates. However, missense mutations could be identified in 9.62% (5/52) of PAS-resistant clinical isolates, and one specific mutation, T120P, could be identified in 5.77% (3/52) of PAS-resistant clinical isolates but not in PAS-sensitive isolates. These data indicate a potential clinical relevance of *rv2172c* and PAS susceptibility, which merits further investigation.

In summary, we identified Rv2172c as an MTHFR in M. tuberculosis and revealed that Arg159 and Leu214 are key catalytic residues for its MTHFR activity. As a result, mutation of the above two residues in Rv2172c caused decreased production of methionine in M. tuberculosis and increased susceptibility to PAS. As expected, *rv2172c* was shown to be essential for the *in vitro* growth of M. tuberculosis, but this essentiality was not limited to methionine biosynthesis. Our data, in combination with previous studies, indicate that Rv2172c might be a good target for designing new anti-TB drugs.

## MATERIALS AND METHODS

### Bacterial strains, plasmids, and culture conditions.

M. tuberculosis H37Ra and derivative strains were cultured at 37°C in 7H9 broth (Difco) supplemented with 10% (vol/vol) oleic acid-albumin-dextrose-catalase (OADC; Difco), 0.5% (vol/vol) glycerol, and 0.05% (vol/vol) Tween 80 (Sigma-Aldrich), or on 7H10 agar medium (Difco) supplemented with 10% (vol/vol) OADC (Difco) and 0.5% (vol/vol) glycerol. M. smegmatis mc^2^155 was grown in Middlebrook 7H9 medium or 7H10 agar medium. E. coli W3110 and derivative strains were cultured in medium E supplemented with 0.5% (vol/vol) dextrose, Luria-Bertani (LB) medium (Difco), or on LB agar plates at 37°C. All bacterial strains, plasmids, and primers used in this study are described in detail in Table S1 in the supplemental material.

### Antibiotics and chemicals.

In total, of 75 μg · mL^−1^ and 150 μg · mL^−1^ hygromycin (Sigma-Aldrich), 25 μg · mL^−1^ and 100 μg · mL^−1^ kanamycin (MD Bio, Inc.), 150 μg · mL^−1^ ampicillin (MD Bio, Inc.), 50 μg · mL^−1^ chloramphenicol (MD Bio, Inc.), and 3.75 to 300 μM methionine (Sigma) were used, unless otherwise indicated. 5,10-Methylenetetrahydrofolate and 5-methyltetrahydrofolate were purchased from Schircks Laboratories. PAS and SMX (Sigma) were used at indicated concentrations.

### 3D structure modeling.

Structural modeling of Rv2172c (UniProtKB accession number O53506) was carried out with the assistance of SWISS-MODEL (40, 41). After searching in the database (https://swissmodel.expasy.org), a suitable template was selected. The structure of the target protein was modeled based on a template in PyMOL, and then graphs were exported for visualization.

### Construction of E. coli and M. tuberculosis mutants and complementary strains.

An unmarked deletion strain of *metF* was constructed as previously described (42). The introduced kanamycin-resistant cassettes were eliminated by transformation with plasmid pCP20. For the construction of complementation strains, genomic DNA from E. coli and M. tuberculosis was extracted. Fragments of *metF* and *rv2172c* were amplified by PCR using specific primers, then ligated into plasmid pCA24N to obtain pCA24N::*metF* and pCA24N::*rv2172c*, respectively. A modified strategy for phage-mediated allelic exchange (43) was used to construct M. tuberculosis H37Ra derivatives containing the derived alleles R159N and L214A. Briefly, *rv2172c* (wild-type) and *rv2172c* alleles were cloned into pMV361 and used to transform M. tuberculosis H37Ra. The native copy of *rv2172c* was deleted by specialized transduction using phAE159 containing a hygromycin resistance cassette. All primers used are listed in Table S1.

### Growth curves.

E. coli W3110 strains and M. tuberculosis strains were cultured to the log phase in medium E containing 0.5% (vol/vol) glucose and separately in 7H9 broth containing OADC at 37°C, then diluted by 10-fold serial dilutions to about 10^6^ CFU · mL^−1^ and 10^5^ CFU · mL^−1^, respectively, in 10 mL fresh medium E with 0.5% glucose and separately in 7H9 broth containing OADC. Then, bacteria were incubated at 37°C. The growth of bacterial cultures was measured by monitoring the optical density at 600 nm (OD_600_) using a spectrophotometer at specified time points (Bio-Rad). Where appropriate, culture medium was supplemented with corresponding levels of methionine. At each time point, a sample of each culture was taken, and serial dilutions were plated on Middlebrook 7H10 plates (for M. tuberculosis) or LB agar plates (for E. coli). The plates were incubated in a normal aerobic atmosphere at 37°C for 2 to 3 weeks (M. tuberculosis) or overnight (E. coli), and the CFU were counted as in a previous study (44). The graphs for growth analysis were prepared using Origin, and the mean values with standard deviations (SDs) were plotted against time.

### Protein purification.

Rv2172c was purified as previously reported (33), with some changes. Briefly, *rv2172c* was amplified from M. tuberculosis H37Ra genomic DNA using specific primers (Table S1) and was cloned into pMAL-c2XHis2 to yield pMAL-c2XHis2::*rv2172c*, which introduced an N-terminal maltose-binding protein (MBP) tag linked with a factor Xa cleavage site and a C-terminal histidine tag. After sequence verification, the recombinant plasmid was transformed into E. coli BL21(DE3). The cells were grown at 37°C in LB broth to an OD_600_ of ∼0.6, isopropyl-β-d-thiogalactopyranoside (IPTG; Acmec) was added to 0.25 mM, and the cells were incubated further at 16°C for 20 h. The bacterial cells were harvested by centrifugation, resuspended in column buffer (CB; 50 mM Tris-HCl and 500 mM NaCl [pH 8.0]) and then disrupted by sonication. Recombinant Rv2172c proteins were first purified over an amylose resin column (product no. E8021; New England Biolabs). To remove the MBP tag, the purified samples were incubated with factor Xa at 4°C overnight in reaction buffer (20 mM HEPES [pH 8.0], 100 mM NaCl, 2 mM CaCl_2_, and 10% glycerol). These cleavage samples were then loaded on prewashed nickel-nitrilotriacetic acid HisTrap HP affinity resin (GE Healthcare) at 4°C overnight, and nonspecifically bound protein was removed by washing this resin with 50 mM Tris-HCl, 500 mM NaCl, and 60 mM imidazole (pH 8.0), while recombinant Rv2172c was eluted with 50 mM Tris-HCl, 500 M NaCl, and 200 mM imidazole (pH 8.0). The fractions were then analyzed by SDS-PAGE.

### *In vitro* enzymatic activity assays.

The MTHFR activity of Rv2172c was measured by HPLC-MS. Five reaction systems (500 μL) were designed to assay purified Rv2172c for its reductase activity *in vitro*. Reaction system 1 contained 100 mM NaH_2_PO_4_, 100 mM Na_2_HPO_4_ (pH 8.0), 2 mM NADH, 100 μM FAD, and 10 mM 5,10-CH_2_-THF. Reaction system 2 contained 100 mM NaH_2_PO_4_, 100 mM Na_2_HPO_4_ (pH 8.0), 10 μM Rv2172c, 2 mM NADH, 100 μM FAD, and 10 mM 5,10-CH_2_-THF. Reaction system 3 contained 100 mM NaH_2_PO_4_, 100 mM Na_2_HPO_4_ (pH 8.0), 10 μM Rv2172c, 100 μM FAD, and 10 mM 5,10-CH_2_-THF. Reaction system 4 contained 100 mM NaH_2_PO_4_, 100 mM Na_2_HPO_4_ (pH 8.0), 10 μM Rv2172c, 2 mM NADH, and 10 mM 5,10-CH_2_-THF. Reaction system 5 contained 100 mM NaH_2_PO_4_, 100 mM Na_2_HPO_4_ (pH 8.0), 10 μM Rv2172c, 2 mM NADH, and 100 μM FAD. All reaction mixtures were incubated at 37°C for 2 h in triplicate, then centrifuged over Microcon-10 protein filter columns at 4°C and 1,000 rpm for 20 min to remove Rv2172c protein. The reaction mixtures were then injected onto a Phenomenex Luna 3-μm C_18_ 100-Å liquid chromatography (LC) column (50 mm × 2 mm) on an UltiMate 3000 ultraperformance liquid chromatography (UPLC) system (Thermo Fisher Scientific). Samples were eluted with a gradient from 90% buffer A (methanol plus 0.1% formic acid) and 10% buffer B (H_2_O plus 0.1% formic acid) to 100% buffer B for 30 min, from 100% buffer B to 100% buffer B for 5 min, and from 100% buffer B to 5% buffer B for 1 min, at a flow rate of 0.3 mL/min. The peaks of the substrate, 5,10-CH_2_-THF, and the product, 5-CH_3_-THF, were measured by UV absorbance (*A*_254_) and confirmed by electrospray ionization-mass spectrometry (ESI-MS) using an in-line LCQ Fleet ion trap mass spectrometer (Thermo Fisher Scientific). The ESI-MS working parameters were as follows: 4.5 kV capillary voltage, 300°C heat block temperature for analysis, and nitrogen drying and nebulizer gases set at 1.5 L/min. The HPLC-MS data were acquired in a scan range between 100 and 1,000 *m/z* using the negative ionization mode.

### Drug susceptibility testing.

Mycobacterial cells were cultured to the mid-log phase (OD_600_ = 0.5 to 1.0) and diluted to about 10^5^ CFU · mL^−1^ using 10-fold serial dilutions in fresh 7H9 medium with or without 10% OADC. Then, bacterial cells were plated on 7H10 solid agar plates containing the following drugs at different concentrations: PAS (0, 0.002, 0.005, 0.01, 0.02, 0.05, 0.1, 0.2, 0.4, 0.8, 1.6, 3.2, and 6.4 μg · mL^−1^) or SMX (0, 0.78125, 1.5625, 3.125, 6.25, 12.5, 25, 50, 100, and 200 μg · mL^−1^). Where appropriate, culture medium was supplemented with 150 μM methionine. All antibiotics were purchased from Sigma-Aldrich and solubilized according to the manufacturer’s recommendations. Cultures were then incubated at 37°C for 21 days. The MIC was defined as the lowest concentration of antibiotics required to inhibit 99% of CFUs after this culture period. The MICs were performed through two technical repetitions using three biological replicates. All of the bacterial strains used are listed in Table S1.

### Antibiotic killing assays.

Bacteria were grown to an OD_600_ of 0.5 to 1.0, diluted to an OD_600_ of ∼0.1 (10^7^ CFU · mL^−1^) in fresh 7H9 medium with OADC, and treated with 2.5 μg · mL^−1^ PAS. Cultures were incubated at 37°C, and aliquots of samples were separately plated on 7H10 medium after serial dilution at days 0, 2, 5, 7, 14, and 21. Where appropriate, culture medium was supplemented with corresponding methionine. Graphs for antibiotic kill curves were prepared using Origin, and the mean values with standard deviations (SDs) were plotted against time.

### Determination of methionine content *in vivo*.

Quantitative detection of l-methionine by UPLC-MS/MS was done using an optimized method previously reported (45). Bacterial samples (∼10^9^ CFU) were resuspended in 0.4 mL precooled methanol-water (2:1, vol/vol) and subjected to three freeze-thaw cycles before sonication in an ice bath for 15 min (cycles: 1-min pulse followed by 1-min pause). The above-described extraction procedure was repeated twice. The mixture was then centrifuged for 10 min at 12,000 × *g* at 4°C, and 10 μL of each supernatant was mixed with 87.5 μL borate buffer (0.2 M, pH 8.8) containing 20 mM Tris(2-carboxyethyl)phosphine hydrochloride (TCEP) and 5 mM ascorbic acid. After vortex mixing and incubation at room temperature for 1 min, the samples were mixed with 33 μL 5-aminoisoquinolyl-*N*-hydroxysuccinimidyl carbamate (5-AIQC) solution and incubated at 55°C for 10 min. The mixture was then cooled down to ambient temperature, and 2 μL of formic acid was added. The mixture was centrifuged for 10 min at 12,000 × *g* at 4°C, and 10 μL of each supernatant was filtered using a 0.22-μm membrane filter before UPLC-MS/MS analysis. UPLC-MS/MS was performed using an Agilent 1290 UPLC instrument coupled to an Agilent 6470 triple quadrupole mass spectrometer equipped with an electrospray ionization (ESI) source (Agilent Technologies, USA). The 5-AIQC-tagged samples (1 μL) were individually injected on an UPLC column (Agilent Zorbax RRHD Eclipse XDB C_18_ column, 2.1 mm × 50 mm, 1.8-μm particles) with the temperature set to 50°C. Water containing 2 mM ammonium bicarbonate and methanol containing 0.16% (vol/vol) formic acid and ammonium formate were used as the two mobile phases A and B, respectively, with a flow rate of 0.5 mL/min. An optimized gradient elution scheme was employed as 5 to 16% B (0 to 2.5 min), 16 to 22.5% B (2.5 to 6 min), 22.5 to 95% B (6 to 8.5 min), and 95% B (8.5 to 12 min). Electrospray ionization was performed using the positive ion mode, the pressure of the nebulizer was 50 lb/in^2^, the sheath gas temperature was 350°C with a flow rate of 10 L/min, the dry gas temperature was 315°C with a flow rate of 10 L/min, and the capillary was set at 4,000 V. Multiple reaction monitoring (MRM) was used for the quantification of screening fragment ions. Peak determination and peak area integration were performed using Mass Hunter Workstation software (version B.08.00; Agilent). Standard curves were constructed by least-squares linear regression analysis using the peak area ratio of derivatized individual standards against the nominal concentration of the calibrator. The quantification of samples was performed identically. The bacterial biomasses of the individual samples were determined by measuring the residual protein content of the metabolite extracts (6). All data obtained by metabolomics were average of independent sextuplicates. *P* values were calculated using *t* tests. The graphs for the determination of methionine *in vivo* were prepared using GraphPad Prism, and median values with 95% confidence levels (CL) were plotted.

### Library preparation and whole-genome sequencing.

Eight individual clinical isolates were provided and characterized as PAS resistant via drug susceptibility testing (DST) by Chongqing Public Health Medical Center (46). Library preparation and whole-genome sequencing were performed at Shanghai Biotechnology Corporation, People’s Republic of China. Genomic DNA of the 8 isolates was extracted from freshly cultured bacteria using the HiPure mycobacteria DNA kit (Magen, Guangzhou, China) following the manufacturer’s protocol. A library was prepared using the NEBNext Ultra DNA library prep kit for Illumina (NEB, USA) following the manufacturer’s protocol and sequenced on a HiSeq 2500 system (Illumina, USA). Library concentration was detected using a Qubit 2.0 fluorometer (Invitrogen, USA) and the Qubit double-stranded DNA (dsDNA) high-sensitivity (HS) kit (Invitrogen, USA). The size and purity of the library were detected using the 2100 Bioanalyzer (Agilent, USA). Raw sequence data were mapped to M. tuberculosis H37Rv reference genome (GenBank accession number AL123456.3) using Bowtie 2 version 2-2.0.5. Single-nucleotide polymorphisms (SNPs) and indels were detected using SAMtools version 1.14 (47).

### Comparative analysis of variants in M. tuberculosis genomes.

As shown in Table S2, a library of M. tuberculosis genomes with the classifications “clinical” (*n* = 6,310) or “environmental” (*n* = 258) was downloaded from the NCBI database. In addition, the genomes of 8 PAS-resistant clinical isolates we recently obtained and genomes of other PAS0resistant or PAS0sensitive clinical isolates (*n* = 44 and 66, respectively) presented in previous research (48–54) are listed in Table S3. All of the raw reads were available and selected after quality inspection. The reference *rv2172c*, *folC*, *thyA*, and *ribD* genes were determined using M. tuberculosis H37Rv (GenBank accession no. NC_000962.3). The homologous genes from other isolates were identified based on the corresponding reference gene using the Basic Local Alignment Search Tool (BLAST) with the default options chosen. All sequences were aligned to the targeted gene fragments using Geneious (v10.2.6). The variation positions were extracted and analyzed using an in-house script.

### Data availability.

All reads generated in this study were deposited in the Sequence Read Archive under accession no. PRJNA774929.
