# Supplementary material for: Decreased Methylenetetrahydrofolate Reductase Activity Leads to Increased Sensitivity to para-Aminosalicylic Acid in Mycobacterium tuberculosis
Source: Antimicrob Agents Chemother. 2022 Jan 18;66(1):e01465-21. doi: 10.1128/AAC.01465-21 (PMC8765232; doi:10.1128/AAC.01465-21)
Supplement: Supplemental file 1 — Table S1, Fig. S1-S3. Download AAC.01465-21-s0001.pdf, PDF file, 0.7 MB [file aac.01465-21-s0001.pdf]

Table S1. Plasmids, strains, and primers used in this study.

Category	Name	Characterization/Sequence (5'-3')	Source
**Plasmids**			
	pMAL-c2XHis2	*E. coli* expression plasmid under control of Tac promoter with N-terminal MBP tag and C-terminal His tag, Ap^R	This study
	pMAL-c2XHis2::*rv2172c*	pMAL-c2XHis2 with *M. tuberculosis* H37Rv *rv2172c* inserted downstream of the Tac promoter, Ap^R	This study
	pCA24N	Expression plasmid under control of T5-lac promoter, Cm^R	Lab stock
	pCA24N::*rv2172c*	pCA24N with *M. tuberculosis* H37Rv *rv2172c* inserted downstream of the T5-lac promoter, Cm^R	This study
	pCA24N::*metF*	pCA24N with *E. coli* MetF inserted downstream of the T5-lac promoter, Cm^R	This study
	pMV261	Non-integrated shuttle plasmid under control of hsp60 promoter, Km^R	Prof. William R Jacobs Jr, Albert Einstein College of Medicine
	pMV261::*rv2172c*	pMV261 with *M. tuberculosis* H37Rv *rv2172c* inserted downstream of the hsp60 promoter, Km^R	This study
	pMV261::*metH*	pMV261 with *M. tuberculosis* H37Rv *metH* inserted downstream of the hsp60 promoter, Km^R	This study
	pMV261::*metE*	pMV261 with *M. tuberculosis* H37Rv *metE* inserted downstream of the hsp60 promoter, Km^R	This study
	pMV361	Integrated shuttle plasmid under control of hsp60 promoter, Km^R	Prof. William R Jacobs Jr, Albert Einstein College of Medicine
	pMV361::*rv2172c*	pMV361 with *M. tuberculosis* H37Rv *rv2172c* inserted downstream of the hsp60 promoter, Km^R	This study
	pMV361::*rv2172c* (R159N)	pMV361 with *M. tuberculosis* H37Rv *rv2172c* (with mutation ^{159}Arg→Asn) inserted downstream of the hsp60 promoter, Km^R	This study
	pMV361::*rv2172c* (L214A)	pMV361 with *M. tuberculosis* H37Rv *rv2172c* (with mutation ^{214}Leu→Ala) inserted downstream of the hsp60 promoter, Km^R	This study
	p0004s	Homologous arms construction plasmid, $HygB^R$	Prof. William R Jacobs Jr, Albert Einstein College of Medicine
	phAE159	Phage transduction plasmid, Ap^R	Prof. William R Jacobs Jr, Albert Einstein College of Medicine
	pKD46	Red recombinase expression plasmids, Ap^R	Yale CGSC
	pKD4	Template plasmids containing a kanamycin resistance gene flanked by FRT sites, Ap^R, Cm^R	Yale CGSC
	pCP20	FLP helper plasmid, Ap^R, Cm^R	Yale CGSC

**Strains**			
	W3110	Wild type for chemotaxis; F- λ- *IN(rrnD-rrnE)1 rph-1*	(1)
	E. coli BL21 (DE3)	Host for protein expression	New England BioLabs
	BL21 (DE3) pMAL-c2XHis2::*rv2172c*	*E. coli* BL21 (DE3) transformed with pMAL-c2XHis2::*rv2172c*	This study
	W3110 pCA24N	*E. coli* W3110 transformed with pCA24N	This study
	W3110 Δ*metF*	*metF* gene deleted in *E. coli* W3110	This study
	W3110 Δ*metF* pCA24N	*E. coli* W3110 Δ*metF* transformed with pCA24N	This study
	W3110 Δ*metF* pCA24N::*metF*	*E. coli* W3110 Δ*metF* transformed with pCA24N::*metF*	This study
	W3110 Δ*metF* pCA24N::*rv2172c*	*E. coli* W3110 Δ*metF* transformed with pCA24N::*rv2172c*	This study
	E. coli HB101	Host for plasmid amplification	Prof. William R Jacobs Jr, Albert Einstein College of Medicine
	M. smegmatis mc^{2}155	High-efficiency transformation strain	Prof. William R Jacobs Jr, Albert Einstein College of Medicine
	H37Ra	*M. tuberculosis* H37Ra avirulent strains	Prof. William R Jacobs Jr, Albert Einstein College of Medicine
	WT	*M. tuberculosis* H37Ra transformed with pMV261	This study
	rv2172c$^+$	*M. tuberculosis* H37Ra transformed with pMV261::*rv2172c*	This study
	metH$^+$	*M. tuberculosis* H37Ra transformed with pMV261::*metH*	This study
	metE$^+$	*M. tuberculosis* H37Ra transformed with pMV261::*metE*	This study
	Rv2172c (WT)	*M. tuberculosis* H37Ra Δ*rv2172c* pMV361::*rv2172c*	This study
	Rv2172c (R159N)	Rv2172c (WT) with mutation *rv2172c* ^{159}Arg→Asn	This study
	Rv2172c (L214A)	Rv2172c (WT) with mutation *rv2172c* ^{214}Leu→Ala	This study
**Primers**			
	Rv2172c-BamHI 261F	AATTGGATCCATGACCCTCAACACGATCGCGCT	This study
	Rv2172c- HindIII 261R	AATTAAGCTTTTAGTCCGGCTTGCCCGGCTCGGC	This study
	metH- EcoRI 261F	AATTGAATTCATGACTGCGGCCGACAAG	This study
	metH- HindIII 261R	GATCAAGCTTTTAACGTTGAAGTACTTGGCTT	This study
	metE- BamHI 261F	AATTGGATCCATGACCCAGCCTGTACGTCGTCAAC	This study
	metE- HindIII261R	AATTAAGCTTTTAGCCCGCGCGCACCTCCCGG	This study
	Rv2172c-EcoRI c2XF	GGAATTCATGACCCTCAACACGATCGCGCT	This study
	Rv2172c-BsmI c2XR	ATAAGAATGCGGCCGCGTCCGGCTTGCCCGGCTCGG	This study
	Rv2172c- BglII 24NF	AATTAGATCTATGACCCTCAACACGATCGCGCT	This study
	Rv2172c- XbalI 24NR	AATTTCTAGACTAGTCCGGCTTGCCCGGCTCG	This study

metF-SacI 24NF	TATAGAGCTCATGAGCTTTTTCACGCCAGCCAGC	This study
metF-PacI 24NR	GGCCTTAATTAATTATAAACCAGGTCGAACCCCCAGC	This study
Rv2172c[a] (R159N)-R	GCACTTGAAATTGAG***ATT***GCCCTGCTCGCCGTC	This study
Rv2172c[a] (R159N)-F	GACGGCGAGCAGGGC***AAT***CTCAATTTCAAGTGC	This study
Rv2172c[a] (L214A)-R	GATCAGCCAGTTGAT***CGC***GCCGATGCGGGTCTC	This study
Rv2172c[a] (L214A)-F	GAGACCCGCATCGGC***GCG***ATCAACTGGCTGATC	This study
Rv2172cko-LFP	TTTTTTTTCCATAAATTGGGCCCACACCCGATGCACTCGCCGCT	This study
Rv2172cko-LRP	TTTTTTTTCCATTTCTTGGGTGGCACCAGCTCCAGCGCGATCGT	This study
Rv2172cko-RFP	TTTTTTTTCCATAGATTGGTAGCTGGTTGGCGGGTCGCCCGAAG	This study
Rv2172cko-RRP	TTTTTTTTCCATCTTTTGGCTGGGTCGCACGCTACCGCTCTAAC	This study
Rv2172c-IDF	TACGCCGAGGTCCGCTCCGAAATGC	This study
Rv2172c-IDR	GCTCAAGGCCGTGGCCTCGGGGAGG	This study
metFko-F	ATGAGCTTTTTCACGCCAGCCAGCGGGATGCCCTGAATCAGAGCCTGGCATATGAATATCCTCCTTAG	This study
metFko-R	TTATAAACCAGGTCGAACCCCCAGCGTATGGCAAATCGCGTAACTCATTTCTGTAGGCTGGAGCTGCTTCG	This study
metF-IDF	TACCGGATTTTCTTTTTCTTACCAT	This study
metF-IDR	AATTAAGCTTTTTTGATTATGTCCG	This study

[a] Sequence which are italic and overstriking are base mutation site.

1. Li Y, Zhang Y. PhoU is a persistence switch involved in persister formation and tolerance to multiple antibiotics and stresses in Escherichia coli. Antimicrob Agents Chemother. 2007;51(6):2092-9.

Supplemental Figures and Figure legends

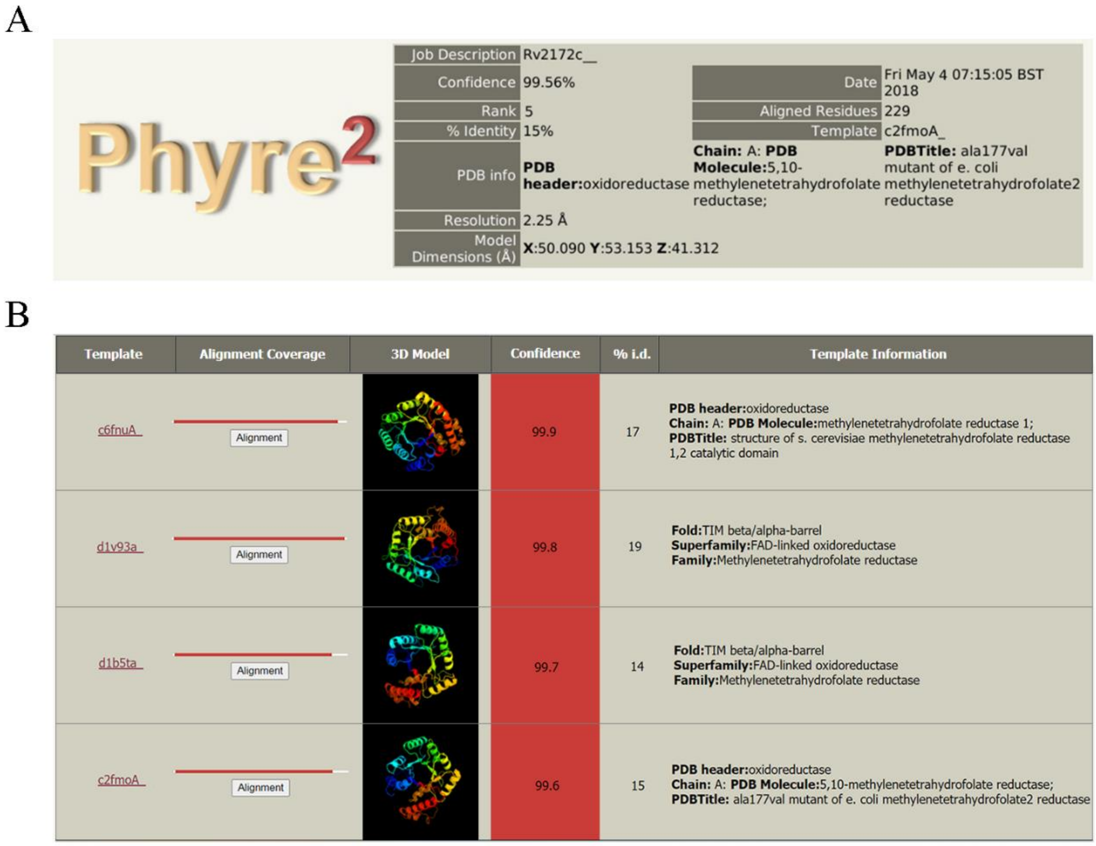

Figure S1. The analysis of Rv2172c by Phyre2. (A) Information on the template *E. coli* MTHFR Ala177Val. (B) Potential 3D models of Rv2172c based on alignment to structure-known templates.

A B

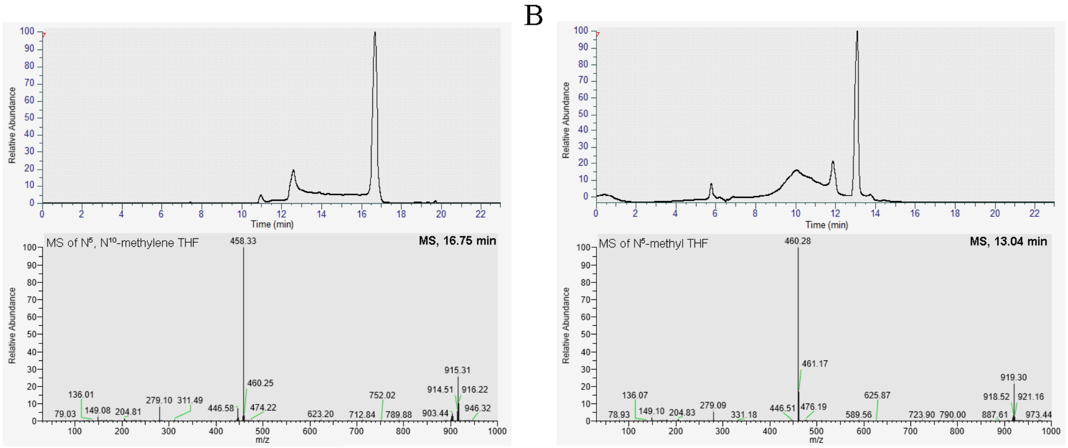

C

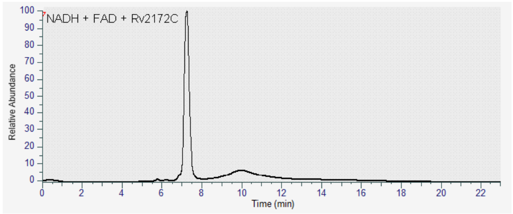

Reaction*	NADH	FAD	Rv2172C	N5, N10-methylene THF (substrate)	N5-methyl THF (product)
1	+	+	-	+	N
2	+	+	+	+	Y
3	-	+	+	+	N
4	+	-	+	+	Y
5	+	+	+	-	N

Figure S2. The supplemental information on reductase activity assays by HPLC-MS. (A) Identification of substrate commercial standard samples. Chromatogram peak at 16.75 min (*top*) and MS peak at 458.33 (*m/z*) (*bottom*) for 5, 10-CH_2-THF. (B) Identification of product commercial standard samples. Chromatogram peak at 13.04 min (*top*) and MS peak at 460.28 (*m/z*) (*bottom*) of 5-CH_3-THF. (C) Chromatogram of no substrate was added in the reaction mixtures of Rv2172c. *Reaction 1, Figure 5A I. Reaction 2, Figure 5A II. Reaction 3, Figure 5A III. Reaction 4, Figure 5A IV. Reaction 5, Figure S2C. N, no detected. Y, detected.

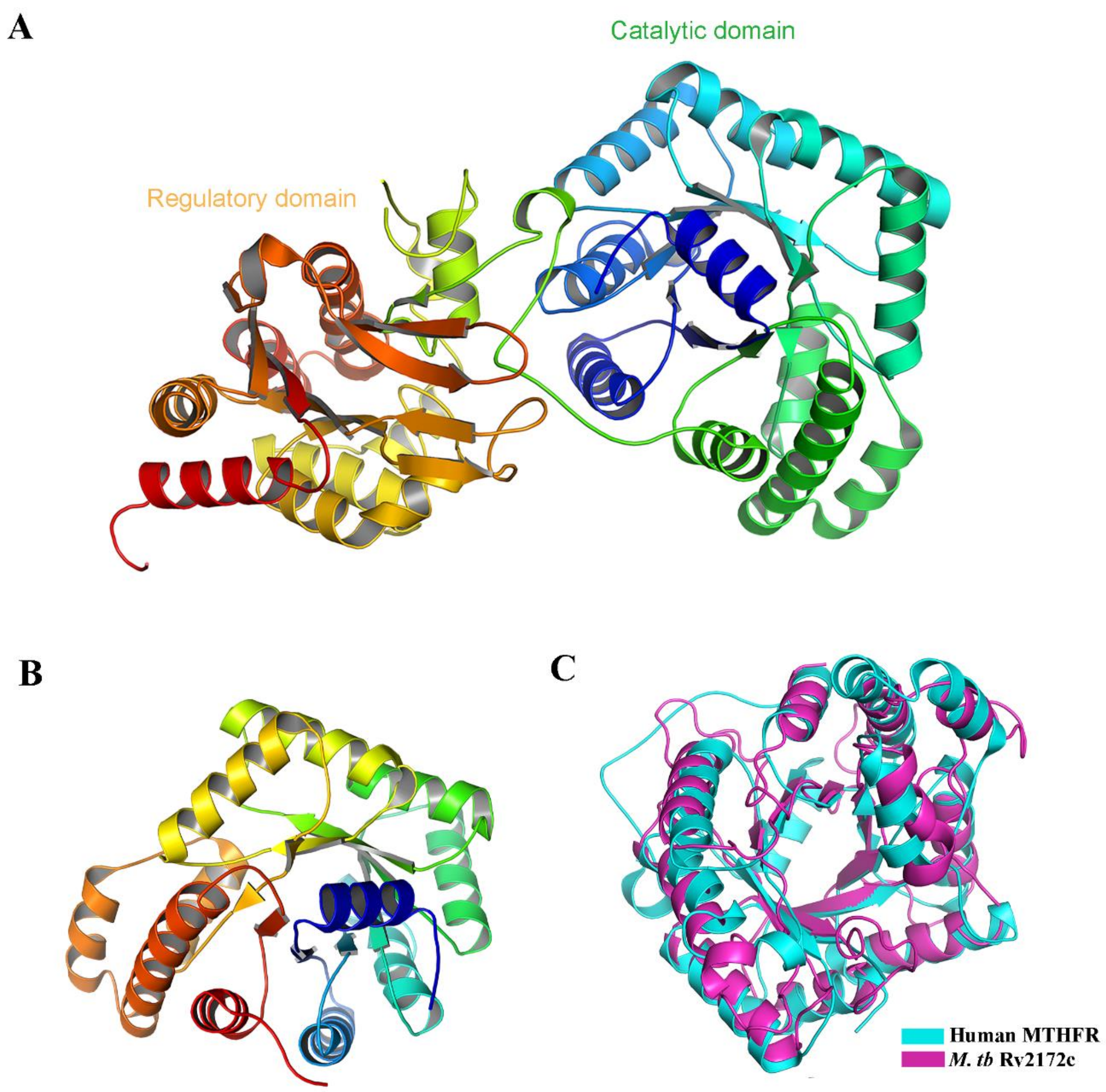

Figure S3. Comparison between the modeling structure of Rv2172c and the known structure of Human MTHFR (PDB entry 6FCX). Rv2172c was modeled based on the best-matched template *Thermus thermophilus* MTHFR (PDB entry 3APT). (A) Overview of Human MTHFR (34) showing the catalytic domain (right), the linker (median), and the regulatory domain (left). (B) Front view of the Human MTHFR (PDB entry 6FCX) catalytic domain. (C) The modeled 3D structure of Rv2172c was superimposed on the Human MTHFR (PDB entry 6FCX) catalytic domain.
